# Efficacy and safety of indacaterol 150 *μ*g once-daily in COPD: a double-blind, randomised, 12-week study

**DOI:** 10.1186/1471-2466-10-11

**Published:** 2010-03-08

**Authors:** Gregory Feldman, Thomas Siler, Niyati Prasad, Damon Jack, Simon Piggott, Roger Owen, Mark Higgins, Benjamin Kramer

**Affiliations:** 1 S. Carolina Pharmaceutical Research, Spartanburg, SC, USA; 2 Midwest Chest Consultants, St Charles, MO, USA; 3 Novartis Horsham Research Centre, Wimblehurst Road, Horsham, West Sussex, UK; 4 Novartis Pharmaceuticals Corp, East Hanover, NJ, USA

## Abstract

**Background:**

Indacaterol is a novel, once-daily (o.d.) inhaled, long-acting *β*_2_-agonist in development for chronic obstructive pulmonary disease (COPD). This 12-week, double-blind study compared the efficacy, safety, and tolerability of indacaterol to that of placebo in patients with moderate-to-severe COPD.

**Methods:**

Efficacy variables included 24-h trough FEV_1 _(mean of 23 h 10 min and 23 h 45 min post-dose) at Week 12 (primary endpoint) and after Day 1, and the percentage of COPD days with poor control (i.e., worsening symptoms). Safety was assessed by adverse events (AEs), mean serum potassium and blood glucose, QTc (Fridericia), and vital signs.

**Results:**

Patients were randomised (n = 416, mean age 63 years) to receive either indacaterol 150 *μ*g o.d. (n = 211) or placebo (n = 205) via a single-dose dry-powder inhaler; 87.5% completed the study. Trough FEV_1 _(LSM ± SEM) at Week 12 was 1.48 ± 0.018 L for indacaterol and 1.35 ± 0.019 L for placebo, a clinically relevant difference of 130 ± 24 mL (p < 0.001). Trough FEV_1 _after one dose was significantly higher with indacaterol than placebo (p < 0.001). Indacaterol demonstrated significantly higher peak FEV_1 _than placebo, both on Day 1 and at Week 12, with indacaterol-placebo differences (LSM ± SEM) of 190 ± 28 (p < 0.001) and 160 ± 28 mL (p < 0.001), respectively. Standardised AUC measurements for FEV_1 _(between 5 min and 4 h, 5 min and 1 h, and 1 and 4 h post-dose) at Week 12 were all significantly greater with indacaterol than placebo (p < 0.001), with LSM (± SEM) differences of 170 ± 24, 180 ± 24, and 170 ± 24 mL, respectively. Indacaterol significantly reduced the percentage of days of poor control versus placebo by 22.5% (p < 0.001) and was also associated with significantly reduced use of rescue medication (p < 0.001). The overall rates of AEs were comparable between the groups (indacaterol 49.3%, placebo 46.8%), with the most common AEs being COPD worsening (indacaterol 8.5%, placebo 12.2%) and cough (indacaterol 6.2%, placebo 7.3%). One patient died in the placebo group. Serum potassium and blood glucose levels did not differ significantly between the two groups, and no patient had QTc >500 ms.

**Conclusions:**

Indacaterol 150 *μ*g o.d. provided clinically significant and sustained bronchodilation, reduced rescue medication use, and had a safety and tolerability profile similar to placebo.

**Trial registration:**

NCT00624286

## Background

Current international clinical practice guidelines for chronic obstructive pulmonary disease (COPD), such as those from the Global Initiative for Chronic Obstructive Lung Disease (GOLD) [1], recommend inhaled bronchodilators, including *β*_2_-agonists and anticholinergics, for the symptomatic management of COPD, with regular use of long-acting bronchodilators having been shown to be more effective and convenient than treatment with short-acting bronchodilators [2-4]. Currently available inhaled long-acting *β*_2_-agonists (LABAs), such as salmeterol and formoterol, provide bronchodilation for approximately 12 h at recommended doses and hence are administered twice daily [5,6]. In chronic diseases such as COPD, compliance to treatment could be improved if the treatment regimens were simplified by reducing dosing frequency [7].

Indacaterol is a novel, once-daily (o.d.), inhaled LABA for the treatment of COPD. Indacaterol is a partial agonist at the human *β*_2_-adrenoceptor with a similar binding affinity to the *β*_2 _receptor as formoterol and higher intrinsic activity than salmeterol [8]. *In vitro *and *in vivo *preclinical studies of this compound have already confirmed its long duration of action (suitable for o.d. dosing in humans), fast onset of action, and improved cardiovascular safety profile [8]. Clinical studies of up to 28 days duration have shown that indacaterol has 24-h bronchodilator efficacy on o.d. dosing, with a good overall safety and tolerability profile [9-11].

The objective of this study was to confirm the efficacy and safety of indacaterol (150 *μ*g), taken o.d. for 12 weeks, in patients with moderate-to-severe COPD.

## Methods

### Study design

This was a 12-week, multi-centre, double-blind, placebo-controlled, parallel-group, Phase III study in patients with moderate-to-severe COPD. The study comprised a pre-screening period, a 2-week screening/run-in period, and a 12-week double-blind treatment period. During the pre-screening visit, patients' ongoing COPD medications were reviewed, and if required, patients were switched from medications prohibited in this study to an allowed COPD therapy (see the Study treatment section). This pre-screening period was followed by a 14-day run-in period (Visits 1 and 2; Day - 14 to Day 1), during which the eligibility of patients for the study was assessed and baseline patient diary data were collected.

The study was conducted in accordance with the Declaration of Helsinki (1989) and local applicable laws and regulations. Approval was obtained from the Institutional Review Board or Independent Ethics Committee of each participating study centre. All patients provided written informed consent prior to participating in the study.

### Study population

Male and female adults (aged ≥40 years) with a clinical diagnosis of COPD (GOLD 2005) [12] and a smoking history of at least 20 pack years were recruited. At Visit 1, patients had to demonstrate a forced expiratory volume in 1 s (FEV_1_) of <80% but ≥30% of the predicted normal value and FEV_1_/forced vital capacity (FVC) of <70% within 30 min of inhalation of 400 *μ*g of salbutamol.

Patients were excluded if they had any recent respiratory tract infection, were hospitalised for a COPD exacerbation (6 weeks prior to Visit 1 or during the run-in period), had a history of asthma (indicated by, but not limited to, blood eosinophil count >400/mm^3 ^or onset of respiratory symptoms prior to age 40 years) or any significant pulmonary disease or cardiovascular abnormality. The following medications were prohibited prior to Visit 1 for at least the minimum washout period (duration is specified within parenthesis) or at any time during the study: long-acting anticholinergic agents (7 days), short-acting anticholinergics (8 h), fixed-dose combination (FDC) of a *β*_2_-agonist and an inhaled corticosteroid (ICS) (48 h), FDC of a short-acting *β*_2_-agonist and a short-acting anticholinergic (8 h), other LABAs (48 h), short-acting *β*_2_-agonists (other than those prescribed in the study) (6 h), xanthine derivatives (1 week), and parenteral or oral corticosteroids (1 month). Patients were also excluded if they were taking nonselective beta-blocking agents, non-potassium sparing diuretics, or certain cardiac antiarrhythmics. Patients on FDCs of a *β*_2_-agonist and an ICS were to be switched to the equivalent ICS prior to the run-in period, with the dose and dosage regimen to remain unchanged for the duration of the study.

### Study treatment

#### Randomisation to treatment groups

Following the screening/run-in period, eligible patients were randomised using validated systems (Visit 3) to receive double-blind indacaterol 150 *μ*g o.d. or matching placebo through a single-dose dry-powder inhaler (SDDPI) for 12 weeks. Randomisation was stratified by smoking status so that the balance was the same in the two treatment groups. Patients were instructed to inhale study medication in the morning (between 08.00 and 11.00 a.m.).

#### Permitted medications

Salbutamol was permitted as a rescue medication throughout the study; however, patients were to refrain from using it within 6 h prior to the study visits. Daily ICS monotherapy was maintained at a constant dose and dosage regimen throughout the study in patients who were previously on ICS or FDC at baseline.

#### Blinding

Patients, investigators, clinical staff performing assessments, data analysts, and the sponsor's trial team were blinded to treatment from the time of randomisation to database lock (unless there were any patient emergencies). All the study drugs were identical in appearance, packaging, labelling, and administration schedule.

### Study assessments

Spirometry measurements were obtained at screening/run-in visits to assess patient eligibility (Day - 14). During the double-blind treatment period, patients' visits to the clinics were scheduled on Days 1, 2, 29, 57, 84, and 85. On Days 1, 29, 57, and 84, FEV_1 _was assessed at 50 and 15 min pre-dose and 5 and 30 min after dose administration in the clinics. On Days 1 and 84, besides the above-mentioned time points, FEV_1 _was also assessed at 1, 2, and 4 h post-dose. Trough FEV_1 _(at 23 h 10 min and 23 h 45 min after the previous day's dose) was measured on Days 2 and 85. Spirometry equipment calibration and spirometric testing were performed in accordance with American Thoracic Society/European Respiratory Society standards [13].

Patients were given a diary to record morning and evening peak expiratory flow (PEF; highest of three consecutive efforts), daily clinical symptoms, rescue salbutamol use, any change in concomitant medications, and adverse events (AEs). Diaries were to be completed at the same time in the morning (before taking study drug) and in the evening (approximately 12 h later). Safety assessments included recording of AEs and serious AEs (SAEs) and monitoring of haematology and blood chemistry (including serum potassium and blood glucose), vital signs, and electrocardiograms including QTc interval (Fridericia's correction). A COPD exacerbation was defined as a new onset or worsening of more than one respiratory symptom (i.e., dyspnoea, cough, sputum purulence or volume, or wheeze) present for more than 3 consecutive days plus either a documented change or increase in COPD-related treatment due to worsening symptoms (e.g., steroids/antibiotics/oxygen), or documented COPD-related hospitalizations or emergency room visits.

#### Variables assessed

The primary efficacy variable was trough FEV_1 _after 12 weeks of treatment (average of the 23 h 10 min and 23 h 45 min post-dose values assessed on the morning of Day 85). The secondary efficacy variables included trough FEV_1 _after one dose (assessed pre-dose on Day 2) and after 29 days, individual time point FEV_1 _on Day 1 and at Week 12 (change from baseline), peak FEV_1 _on Day 1 and at Week 12 (assessed between 5 min and 4 h post-dose), and standardised area under the curve (AUC) for FEV_1 _between 5 min and 4 h, 5 min and 1 h, and 1 h and 4 h at Week 12.

Other efficacy outcomes were based on data recorded on patients' diaries. These included use of rescue medication, PEF, and the number of 'days of poor control', defined as any day in the patient diary in which a score ≥2 (i.e., moderate or severe symptoms) was recorded for at least two out of five symptoms (cough, wheeze, production of sputum, colour of sputum, and breathlessness). This instrument has not been validated, although it was used previously in the Foradil registration programme [4,6].

### Sample size and statistical analyses

A difference of 120 mL in trough FEV_1 _between indacaterol and placebo was pre-specified as the minimal clinically important difference for this population. A standard deviation of 270 mL was assumed for trough FEV_1_, based on the results from previous formoterol studies [6,8]. It was calculated that, allowing for a potential imbalance between treatment groups, a sample size of 232 evaluable patients would be needed to test for the minimal clinically important difference of 120 mL between indacaterol 150 *μ*g and placebo at the (two-sided) 5% significance level with 90% power. Allowing for a drop-out rate of 20%, it was estimated that a minimum of 290 patients were needed to provide 90% power for the primary endpoint.

All efficacy analyses were performed on the intent-to-treat (ITT) population, which included all patients who were randomised to receive treatment. All safety analyses were performed on the safety population, which included all patients who received at least one dose of the study medication.

A mixed model was used to analyse the primary variable (imputed with last observation carried forward) with treatment as a fixed effect and the baseline FEV_1 _and FEV_1 _reversibility components as covariates. The model also included smoking status (current/ex-smoker) and country as fixed effects, with centre nested within country as a random effect. The least squares means (LSM), i.e., means adjusted for the covariates in the model, of the treatment contrast for indacaterol 150 *μ*g versus placebo was estimated along with the associated 95% confidence interval and two-sided p-value, and superiority of indacaterol over placebo was demonstrated only if the p-value was less than the pre-specified 5% significance level.

## Results

### Patients

**Figure 1:**
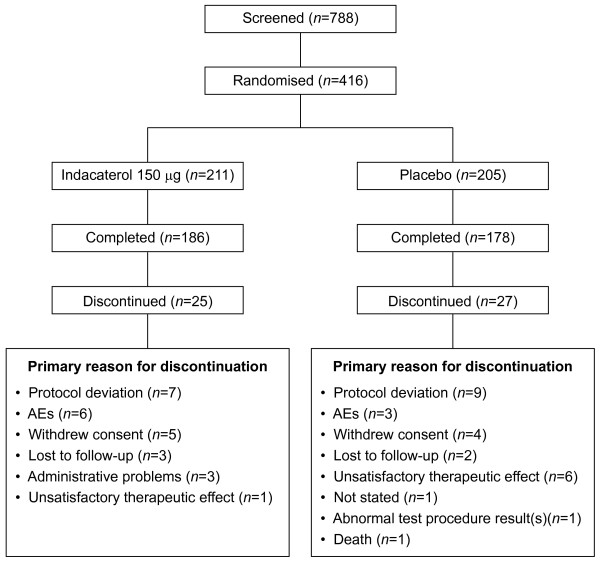
**Patient disposition**.

This study was conducted at 103 centres in 3 countries (US, New Zealand and Belgium). A total of 788 patients were screened, with 416 randomised to either indacaterol 150 *μ*g (n = 211) or placebo (n = 205). Overall, 364 patients (87.5%) completed the study, (Figure 1). Both treatment groups were comparable and well matched with respect to baseline demographic and disease characteristics (Table 1); 52.4% of patients were male and 92.5% were Caucasian. The mean duration of COPD was 6.9 years, with diagnosis status ranging from newly diagnosed to 38.7 years of disease history.

**Table 1:** Patient demographic and baseline clinical characteristics (safety population)

	Indacaterol 150 *μ*g o.d. N = 211	Placebo N = 205
Age, in years, mean (SD)	62.9 (9.89)	63.2 (9.62)

Sex, n (%)		

Male	108 (51.2)	110 (53.7)

Female	103 (48.8)	95 (46.3)

Race, n (%)		

Caucasian	194 (91.9)	191 (93.2)

Black	12 (5.7)	10 (4.9)

Asian	1 (0.5)	1 (0.5)

Native American	2 (0.9)	1 (0.5)

Other	2 (0.9)	2 (1.0)

Duration of COPD, in years, mean (SD)	6.6 (6.86)	7.3 (5.64)

Duration of COPD, in years, n (%)		

<1	38 (18.0)	18 (8.8)

1—5	74 (35.1)	69 (33.7)

>5—10	57 (27.0)	69 (33.7)

>10—15	23 (10.9)	30 (14.6)

>15—20	9 (4.3)	12 (5.9)

>20	10 (4.7)	7 (3.4)

Severity of COPD*, n (%)		

At risk	0 (0.0)	1 (0.5)

Mild	7 (3.3)	10 (4.9)

Moderate	119 (56.4)	117 (57.1)

Severe	84 (39.8)	76 (37.1)

Very severe	1 (0.5)	1 (0.5)

Smoking history, n (%)		

Ex-smoker	103 (48.8)	97 (47.3)

Current smoker	108 (51.2)	108 (52.7)

Number of pack years^†^, mean (SD)	53.5 (26.84)	60.5 (54.12)

FEV_1 _(L), mean (SD)^‡^	1.5 (0.53)	1.5 (0.51)

FEV_1 _(% predicted), mean (SD)^‡^	54.4 (13.38)	55.8 (14.08)

FEV_1_/FVC (%), mean (SD)^‡^	53.5 (9.84)	53.5 (10.36)

FEV_1 _reversibility (%), mean (SD)	16.4 (17.31)	16.6 (19.44)

Concomitant ICS, n (%)	61 (28.9)	70 (34.1)

COPD = chronic obstructive pulmonary disease; FEV_1 _= forced expiratory volume in 1 s; FVC = forced vital capacity; ICS = inhaled corticosteroids.

*As per GOLD 2005

^†^Pack years = total years of smoking multiplied by cigarette packs smoked per day

^‡^Post-bronchodilator (within 30 min after inhaling 400 *μ*g salbutamol)

Almost all randomised patients (99.0%) had at least one active medical condition, the most common being hypertension (51.4%), gastro-oesophageal reflux (30.0%), depression (23.8%), osteoarthritis (22.4%), and hyperlipidaemia (21.6%). Mean patient exposures to study medication were similar between treatment groups (85.0 and 84.0 days for indacaterol and placebo, respectively), and compliance was high in both groups (>97%).

### Efficacy

#### Spirometry evaluations

**Figure 2:**
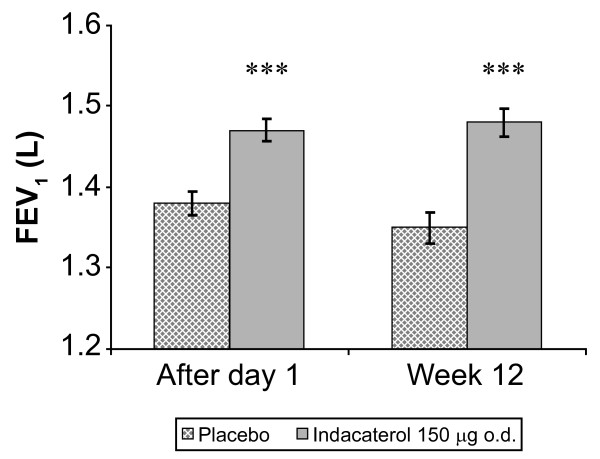
**24-h post-dose (trough) FEV_1 _after 1 day and at Week 12 of treatment (ITT population)**. Data are LSM ± SEM. Significant treatment difference: ***p < 0.001 versus placebo. FEV_1 _= forced expiratory volume in 1 s; ITT = intent-to-treat; LSM = least squares means; SEM = standard error of mean.

For the primary endpoint, that is, 24-h post-dose trough FEV_1 _at Week 12, indacaterol provided a bronchodilator efficacy superior to that of placebo, with an LSM ± standard error of the mean (SEM) indacaterol-placebo difference of 130 ± 24 mL (p < 0.001), which exceeded the 120 mL threshold for clinical relevance (Figure 2). Indacaterol also provided statistically superior efficacy to placebo for 24-h post-dose trough FEV_1 _after the first dose, with a difference from placebo of 80 ± 19 mL (p < 0.001) (Figure 2). On Day 29, the indacaterol-placebo difference was 140 ± 24 mL (p < 0.001).

**Figure 3:**
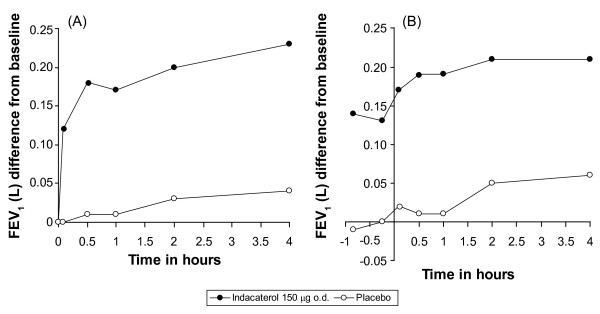
**Individual time-point FEV_1 _on Day 1 and at Week 12 (change from baseline, ITT population)**. Individual time-point FEV_1 _(A) on Day 1 (baseline to 4 h) and (B) at Week 12 (-50 min to +4 h). FEV_1 _= forced expiratory volume in 1 s; ITT = intent-to-treat.

Individual time-point FEV_1 _(mean change from baseline) over the first 4 h post-dose on Day 1 and at Week 12 are shown in Figure 3. At all post-baseline time points, indacaterol provided statistically superior FEV_1 _to that of placebo (p < 0.001). At the first post-baseline time point assessed (5 min post-dose on Day 1), the LSM ± SEM indacaterol-placebo difference was 130 ± 15 mL (p < 0.001).

Indacaterol 150 *μ*g o.d. was associated with significantly higher peak FEV_1 _than placebo, both on Day 1 and at Week 12, with LSM ± SEM indacaterol-placebo differences of 190 ± 28 mL (p < 0.001) and 160 ± 28 mL (p < 0.001), respectively. Furthermore, standardised AUC measurements for FEV_1 _(between 5 min and 4 h, 5 min and 1 h, and 1 and 4 h post-dose) at Week 12 were all significantly greater with indacaterol 150 *μ*g as compared with placebo (p < 0.001), with LSM differences of 170 ± 24 mL, 180 ± 24 mL, and 170 ± 24 mL, respectively (Table 2).

**Table 2:** Standardised AUC for FEV_1 _at Week 12: treatment comparisons (ITT population)

Treatment		Treatment difference
	LSM ± SEM	LSM ± SEM

AUC_(5 min—4 h) _for FEV_1_		

Indacaterol 150 *μ*g o.d.	1.54 ± 0.019	
		0.17 ± 0.024*
Placebo	1.37 ± 0.020	

AUC_(5 min—1 h) _for FEV_1_		

Indacaterol 150 *μ*g o.d.	1.52 ± 0.020	
		0.18 ± 0.024*
Placebo	1.34 ± 0.020	

AUC_(1 h—4 h) _for FEV_1_		

Indacaterol 150 *μ*g o.d.	1.55 ± 0.020	
		0.17 ± 0.024*
Placebo	1.37 ± 0.021	

*p < 0.001

AUC = area under the curve; FEV_1 _= forced expiratory volume in 1 s; ITT = intent-to-treat; LSM = least squares mean; SEM = standard error of mean

#### Patient diary evaluations

Patients were asked to use their diaries twice daily to record their PEF, rescue medication use, and symptoms. Over the 12 weeks of the study, the changes from baseline in both morning and evening PEF were significantly greater for indacaterol 150 *μ*g versus placebo, with LSM ± SEM improvements versus placebo of 24.6 ± 3.18 and 23.6 ± 3.11 L/min in morning and evening PEF, respectively (both, p < 0.001). In addition, patients taking indacaterol 150 *μ*g o.d. required significantly less rescue medication compared with patients on placebo, as measured by mean daily, daytime, and night-time number of puffs of rescue medication (all, p < 0.001), with a significant improvement in the percentage of days with no rescue use (p < 0.001; Table 3). The percentage of COPD 'days of poor control' over 12 weeks of treatment (defined in the Methods section) was 22.5% lower in the indacaterol 150 *μ*g o.d. treatment group compared with the placebo group (p < 0.001; Table 3).

**Table 3:** Rescue medication use and percentage of COPD 'days of poor control' over 12 weeks of treatment (ITT population)

	Treatment	Treatment difference
	**LSM ± SEM**	**LSM ± SEM**

**Change from baseline in the mean daily number of puffs of rescue medication**		

Indacaterol 150 *μ*g o.d.	-1.38 ± 0.118	
		-0.97 ± 0.168*
Placebo	-0.41 ± 0.122	

**Change from baseline in the mean day time number of puffs of rescue medication**		

Indacaterol 150 *μ*g o.d.	-0.92 ± 0.076	
		-0.65 ± 0.109*
Placebo	-0.27 ± 0.079	

**Change from baseline in the mean night time number of puffs of rescue medication**		

Indacaterol 150 *μ*g o.d.	-0.47 ± 0.052	
		-0.34 ± 0.075*
Placebo	-0.13 ± 0.054	

**Percentage of days with no rescue use**		

Indacaterol 150 *μ*g o.d.	54.63 ± 1.942	
		13.35 ± 2.791*
Placebo	41.28 ± 2.004	

**Percentage of COPD 'days of poor control'**		

Indacaterol 150 *μ*g o.d.	31.19 ± 1.500	
		-9.05 ± 2.175*
Placebo	40.24 ± 1.554	

COPD = chronic obstructive pulmonary disease; ITT = intent-to-treat;

LSM = least squares mean; SEM = standard error of the mean

* p < 0.001

### Safety

The overall rate of AEs was comparable between the two treatment groups (Table 4). The most frequently reported AEs in the indacaterol and placebo groups by preferred term were COPD worsening (including exacerbations; 8.5% and 12.2%, respectively) and cough (6.2% and 7.3%, respectively). The incidences of AEs known to be *β*_2_-agonist class effects (muscle spasm, headache, and tremor) were comparable between the indacaterol and placebo groups. Most of the AEs observed were of mild or moderate intensity; severe AEs occurred in 4.7% of indacaterol-treated patients versus 5.4% of placebo-treated patients. AEs suspected to be related to study drug occurred in a similar proportion of patients in the two treatment groups (8.1% in the indacaterol vs. 7.8% in the placebo group). One patient in the placebo group died during the study (female, 51 years). The cause of death was unknown at the time but was suspected to be related to study medication by the sponsor's medical safety physician in the absence of the investigator's causality assessment. However, an autopsy suggested that the cause of death was cardiac arrhythmia due to cardiomegaly, and the investigator assessed the event as not related to study medication. None of the other SAEs reported were considered to be related to study medication.

**Table 4:** Adverse events (including COPD exacerbations) overall and by primary system organ class (>3% in either treatment group; Safety population)

	Indacaterol 150 *μ*g o.d. N = 211 n (%)	Placebo N = 205 n (%)
**Patients with any adverse event (s)**	104 (49.3)	96 (46.8)

**Primary system organ class**		

Respiratory, thoracic and mediastinal disorders	40 (19.0)	43 (21.0)

Infections and infestations	37 (17.5)	28 (13.7)

Gastrointestinal disorders	19 (9.0)	14 (6.8)

Musculoskeletal and connective tissue disorders	16 (7.6)	17 (8.3)

Nervous system disorders	11 (5.2)	11 (5.4)

General disorders and administration site conditions	9 (4.3)	10 (4.9)

Injury, poisoning and procedural complications	9 (4.3)	5 (2.4)

Investigations	7 (3.3)	7 (3.4)

COPD = chronic obstructive pulmonary disease

Investigators were also asked to record any instances of cough occurring within 5 min of drug administration during study visits, regardless of whether they considered this to be an AE. This was observed at clinic visits with an average incidence of 17.8% with indacaterol and 3.3% with placebo. The onset of cough following inhalation was predominantly within 15 s of inhalation, with median duration of 6 s, and there was no association between cough and bronchospasm. Importantly, the presence of this cough was not associated with any increase in study discontinuation rates.

Laboratory evaluations (including blood glucose and serum potassium) showed no clinically relevant differences between the indacaterol 150 *μ*g and placebo groups. No patient had a clinically notable low potassium value (i.e., <3.0 mmol/L), while the proportion of patients with clinically notable hyperglycaemia (>9.99 mmol/L) was lower in the indacaterol group than in the placebo group (3.3% vs. 6.3%).

The percentage of patients who had a maximum post-baseline pulse rate >90 bpm at any visit was lower in the indacaterol group as compared with the placebo group (5.7% vs. 11.2%), and no patients in either group experienced either a pulse rate >130 bpm or a pulse rate ≥120 bpm with a change from baseline ≥15 bpm. No patients in the indacaterol group experienced a maximum post-baseline systolic blood pressure >200 mmHg, or ≥180 mmHg with an increase from baseline ≥20 mmHg (compared with one patient receiving placebo); one patient in the indacaterol group (compared with none in the placebo group) had a maximum post-baseline diastolic blood pressure ≥105 mmHg with an increase from baseline ≥15 mmHg. The number of patients with a notable maximum post-baseline QTc interval (Fridericia's; i.e., >450 ms for males or >470 ms for females) was 7 (3.3%) in the indacaterol versus 4 (2.0%) in the placebo group. No patient in either group had a QTc interval (Fridericia's) >500 ms and none had an increase from baseline in QTc interval of >60 ms.

## Discussion

This study was designed to assess the 12-week efficacy and safety of indacaterol 150 *μ*g o.d. in patients with moderate-to-severe COPD. Significant bronchodilation was observed following administration of the first dose of indacaterol, with efficacy sustained over the full 12-week treatment period. Trough FEV_1 _after 12 weeks of treatment (the primary endpoint) exceeded the placebo value by more than 120 mL (the pre-specified minimum clinically important difference). This value of 120 mL is higher than the 100 mL described by Donohue as a difference that patients can perceive [14] and is the midpoint of the 100-140 mL range proposed recently as a minimal clinically important difference [15]. A statistically superior improvement in FEV_1 _for indacaterol versus placebo was also observed at all individual post-baseline time points on Day 1 and Week 12, with improvements versus placebo for FEV_1 _AUCs between 5 min and 1 h, 5 min and 4 h, and 1 and 4 h post-dose. These results demonstrate a sustained 24-h duration of action of indacaterol on o.d. dosing. This persistence of treatment effect has also been observed in other published indacaterol studies [9-11], including a double-blind crossover study in patients with moderate-to-severe COPD, in which a single dose of indacaterol 150 *μ*g provided comparable 24-h trough FEV_1 _to twice-daily formoterol [11].

Although a sustained 24-h effect is advantageous in the treatment of COPD patients, it is important that this is not accompanied by a loss in efficacy on chronic dosing (or tolerance). *β*_2_-adrenoceptor downregulation following chronic dosing with LABAs may result in the development of tolerance to the bronchodilatory effects of LABAs [16], and previous studies have shown the diminution in efficacy over time in other bronchodilators, including salmeterol [17]. The results of our study demonstrate that there was no loss in efficacy over the 12 weeks of treatment, with indacaterol-placebo differences maintained from Day 29 (the first trough assessment after indacaterol is known to have reached steady-state) to Week 12 in terms of trough FEV_1_. These data are consistent with other previously published studies with indacaterol, in which there was no loss in efficacy on daily dosing for up to a year. For example, in a 1-year study, the trough FEV_1 _for indacaterol 150 *μ*g was 160 mL higher than placebo after 12 weeks of dosing, compared with 170 mL after a year [18].

In the current study, serial measurements of FEV_1 _on Day 1 also showed that indacaterol 150 *μ*g o.d. provided statistically significant (compared to placebo) and clinically relevant bronchodilation at 5 min post-dose, the earliest post-dose assessment time point in this study, and is consistent with previous studies [10,11]. The o.d. dosing regimen of indacaterol, combined with clinically relevant bronchodilation at 5 min post-dose may contribute to improved adherence to the prescribed dose regimen in the 'real world' setting—an important consideration, because adherence to COPD medication in clinical practice are reported to be between 10 and 40% [19-21].

Alongside the improvements in FEV_1_, indacaterol also provided improvements compared with placebo in terms of a range of data captured by patients in their daily diaries, including significant improvements from baseline in morning and evening PEF, and reductions in the requirement for rescue medication. The frequency of use of rescue medication for symptomatic relief provides an indication of the degree of patient impairment. It is, therefore, of note that the use of indacaterol was associated with an overall reduction in rescue medication use, with improvements observed both in daytime and night-time over a 24-h dosing interval.

Indacaterol 150 *μ*g o.d. was well tolerated, with a high level of treatment compliance and a low drop-out rate. The overall incidence of AEs was comparable between indacaterol and placebo, and AEs were mostly mild and transient, with the majority being typical of this patient population. The most frequently reported AEs were COPD worsening and cough, the incidences of which were lower in the indacaterol group than placebo. The presence of cough following soon after inhalation was not associated with any increase in study discontinuation rates, and there was no suggestion of a relationship between these cough events and cough reported as an AE. The class-related side effects of *β*_2_-agonists (e.g., hyperglycaemia, hypokalaemia, or prolonged QTc interval) were low in overall incidence in this study. No patient had a clinically notable low potassium value (i.e. <3.0 mmol/L), while clinically notable hyperglycaemia (>9.99 mmol/L) occurred in fewer patients in the indacaterol group than in the placebo group. No patient in either group had an absolute QTc interval (Fridericia's) of greater than 500 ms, and none had an increase in QTc from baseline of more than 60 ms. Cardiac safety is of particular concern in COPD patients considering the fact that cardiovascular comorbidity is common in these patients; therefore, the absence of detrimental cardiovascular incidents in this study, together with results from earlier shorter-duration studies, suggest that the risk of adverse cardiovascular effects with indacaterol 150 *μ*g is low [9-11]. Moreover, data from other studies have shown similar safety findings with single doses of up to 3000 *μ*g [22].

## Conclusions

Indacaterol 150 *μ*g o.d. showed effective bronchodilation in patients with moderate-to-severe COPD, with a significantly reduced rescue medication use compared with placebo. Our results suggest that indacaterol may present a useful alternative to the currently available twice-daily LABAs, given the sustained 24-h bronchodilation on o.d. dosing.

## Competing interests

GF and TS have been reimbursed by Novartis, the sponsors of this clinical trial, for attending

Conferences. BK is an employee of Novartis and has shares in the Company. NP, DJ, SP, RO, and MH are employees of Novartis.

## Authors' contributions

All the authors contributed equally to this work. GF was the principal investigator of the study and approved the interpretation of data and the study report. GF and TS contributed to the acquisition of study data. Novartis (represented by DJ, NP, SP, RO, MH and BK) was responsible for the conception and design of the study, and analysis and interpretation of data. All authors had access to the study data, contributed equally to the development of the submitted article, revising it critically for important intellectual content, and had the responsibility of final approval before submission for publication.

## Note

†**IN**dacaterol efficacy eva**L**uation us**I**n**G **150 *μ*g doses wit**H **COPD pa**T**ients 1

## Pre-publication history

The pre-publication history for this paper can be accessed here:

http://www.biomedcentral.com/1471-2466/10/11/prepub
